# Immune Activation in Functional Dyspepsia: Bystander Becoming the Suspect

**DOI:** 10.3389/fnins.2022.831761

**Published:** 2022-04-26

**Authors:** Matthias Ceulemans, Inge Jacobs, Lucas Wauters, Tim Vanuytsel

**Affiliations:** ^1^Department of Chronic Diseases and Metabolism, Translational Research Center for Gastrointestinal Disorders (TARGID), Katholieke Universiteit Leuven, Leuven, Belgium; ^2^Allergy and Clinical Immunology Research Group, Department of Microbiology, Immunology and Transplantation, Katholieke Universiteit Leuven, Leuven, Belgium; ^3^Department of Gastroenterology and Hepatology, University Hospitals Leuven, Leuven, Belgium

**Keywords:** functional dyspepsia, immune activation, disorders of gut brain interaction, eosinophil, mast cell, functional gastrointestinal disorders, gut-brain axis, irritable bowel syndrome

## Abstract

Disorders of gut-brain interaction (DGBI), formerly termed functional gastrointestinal disorders (FGID), are highly prevalent although exact pathophysiological mechanisms remain unclear. Intestinal immune activation has been recognized, but increasing evidence supports a pivotal role for an active inflammatory state in these disorders. In functional dyspepsia (FD), marked eosinophil and mast cell infiltration has been repeatedly demonstrated and associations with symptoms emphasize the relevance of an eosinophil-mast cell axis in FD pathophysiology. In this Review, we highlight the importance of immune activation in DGBI with a focus on FD. We summarize eosinophil biology in both homeostasis and inflammatory processes. The evidence for immune activation in FD is outlined with attention to alterations on both cellular and molecular level, and how these may contribute to FD symptomatology. As DGBI are complex and multifactorial conditions, we shed light on factors associated to, and potentially influencing immune activation, including bidirectional gut-brain interaction, allergy and the microbiota. Crucial studies reveal a therapeutic benefit of treatments targeting immune activation, suggesting that specific anti-inflammatory therapies could offer renewed hope for at least a subset of DGBI patients. Lastly, we explore the future directions for DGBI research that could advance the field. Taken together, emerging evidence supports the recognition of FD as an immune-mediated organic-based disorder, challenging the paradigm of a strictly functional nature.

## Introduction

Functional dyspepsia (FD) is a chronic gastrointestinal (GI) disorder affecting nearly 10% of the population as surveyed by internet questionnaires and personal interviews worldwide (Sperber et al., 2021a). FD can have a substantial impact on patients’ quality of life and their ability to perform daily activities (Talley et al., 2006). The combination of a decreased work productivity, a lack of cost-effective treatment options and high health-care costs also imply a substantial economic burden (Moayyedi and Mason, 2002; Lacy et al., 2013).

Cardinal symptoms of FD include postprandial fullness and early satiation, both meal-related symptoms that identify the subgroup of postprandial distress syndrome (PDS). Patients suffering from painful symptoms such as epigastric pain and burning are classified as epigastric pain syndrome (EPS) subtype (Stanghellini et al., 2016). According to the latest iteration of the Rome consensus on diagnostic criteria for FD (Rome IV), an overlap between both subgroups is categorized as a separate entity, although postprandial complaints – whether or not pain-related – should be regarded as part of PDS (Stanghellini et al., 2016). Moreover, the diagnosis of FD is based on exclusion of any structural abnormality found during routine diagnostic tests including gastroduodenoscopy, or any organic cause that could explain symptoms (Stanghellini et al., 2016).

Although the presence of major structural alterations is not in line with the current Rome IV diagnostic criteria, microscopic duodenal alterations have become increasingly recognized as a part of FD pathophysiology. Low-grade immune activation in the duodenum, manifested by increased mucosal eosinophil and mast cell numbers, was associated with increased permeability of the duodenal epithelium (Vanheel et al., 2014), challenging the paradigm of FD as a merely functional disorder. The underlying pathophysiological mechanisms are likely multifactorial, but still poorly understood, which complicates the management of individual patients.

Known risk factors for FD are female gender, anxiety and use of non-steroidal anti-inflammatory drugs or antibiotics (Wauters et al., 2021e). Other factors potentially linked to FD pathogenesis include both luminal (e.g., microbial components, food antigens or secreted factors such as acid and bile), and central triggers such as stress or disordered gut-brain communication, leading to disturbances in GI motility and sensitivity. Hence, FD is termed a disorder of gut-brain interaction (DGBI) (Drossman and Hasler, 2016), until recently mainly known as functional gastrointestinal disorder (FGID).

In this Review, we provide a narrative overview on immune activation in DGBI with a focus on FD and particular attention for the role of eosinophils in homeostasis and inflammation. We also discuss various factors associated to immune activation and how they can contribute to FD pathophysiology, before we address overlapping symptomatology and inflammatory differences with other DGBI. Finally, we discuss the evidence for anti-inflammatory therapies in FD and explore future directions in the field.

## Intestinal Mucosal Eosinophils In Homeostasis And Inflammation

Eosinophils are pleiotropic leukocytes present under homeostatic conditions throughout the GI tract, with an exception for the esophagus in health, which is generally devoid of eosinophils (Kato et al., 1998). Eosinophils differentiate from pluripotent hematopoietic progenitor cells in the bone marrow under influence of the transcription factor GATA-1 and eosinophilopoietins including granulocyte-macrophage colony stimulating factor (GM-CSF), interleukin (IL)-3 and -5 (Rothenberg and Hogan, 2006). The latter drives the maturation and proliferation of eosinophils as well as eosinophil entry into the blood stream (Collins et al., 1995; Rothenberg and Hogan, 2006). The importance of IL-5 in eosinophil biology is highlighted as its expression was found to be sufficient for induction of both systemic and multi-organ eosinophilia in murine models (Sanderson, 1992). Similar to T-lymphocytes, eosinophils rely on the expression of surface integrins α_4_β_7_ in order to migrate into the small intestine and colon (Brandt et al., 2006). Besides, eosinophil recruitment to the intestinal mucosa is also regulated by the expression of CC chemokine receptor (CCR)3, which binds members of the eotaxin family (Daugherty et al., 1996; Ponath et al., 1996). Eotaxin-1 (CC chemokine ligand (CCL)11) is constitutively expressed in the GI tract to attract mature eosinophils (Matthews et al., 1998; Rothenberg, 1999; Rothenberg and Hogan, 2006). Other potent eosinophil chemoattractants include eotaxin-2 (CCL24), eotaxin-3 (CCL26), regulated upon activation, normal T cell expressed and secreted (RANTES, CCL5), monocyte chemoattractant protein (MCP)-2, -3, and -4 (Luster and Rothenberg, 1997; Weller and Spencer, 2017). Among these, eotaxin-1 is thought to be the most important eosinophil chemoattractant in health (Matthews et al., 1998; Powell et al., 2010) and seems to regulate eosinophil chemotaxis mainly in the lower GI tract, whereas eotaxin-3 is suggested to be more involved in the proximal GI tract (Blanchard and Rothenberg, 2009).

Mucosal eosinophil numbers are thought to increase progressively from the proximal small intestine to the cecum as reported by pediatric studies (Lowichik and Weinberg, 1996; DeBrosse et al., 2006; Kiss et al., 2018; Koutri et al., 2020), although an opposite gradient is found in mice (Mowat and Agace, 2014; Brown and Esterházy, 2021). Eosinophils represent around 20% of the leukocyte population in the lamina propria of the proximal small intestine (Jung and Rothenberg, 2014). In this region, the innate immune profile appears to be predisposed toward a T-helper (Th)2 phenotype, rather than Th1 or -17 (Brown and Esterházy, 2021). Eosinophils are classically thought of as innate effector cells in a Th2 initiated response to invading pathogens such as helminths. Th2-lymphocyte released IL-4, -5 and -13 can activate eosinophils *via* eotaxins in a signal transducer and activator of transcription (STAT)6-dependent mechanism (Zimmermann et al., 2003). Increasing evidence supports the role of group 2 innate lymphoid cells (ILC2) as important producers of Type 2 cytokines including IL-5 and -13, thereby contributing to eosinophil recruitment (Nussbaum et al., 2013; Larose et al., 2017). Eosinophil activation results in degranulation with release of various cytotoxic granular proteins including major basic protein (MBP), eosinophil cationic protein (ECP), eosinophil peroxidase (EPO) and eosinophil-derived neurotoxin (EDN) (Rothenberg and Hogan, 2006). However, recent findings increasingly point toward an important contribution of eosinophils to GI homeostasis (Shah et al., 2020; Masterson et al., 2021; Wechsler et al., 2021). These novel roles include promoting the survival and class switching of immunoglobulin (Ig)A-producing plasma cells, supporting IL-1β-mediated IgA production, maintaining Th17 cell differentiation and supporting microbial homeostasis, mucus production and inflammatory homeostasis in the intestinal microenvironment (Jung et al., 2015; Marichal et al., 2017).

Eosinophils are typically involved in allergic conditions such as asthma, atopic dermatitis and rhinitis, but increased infiltration of these cells is also observed in multiple GI disorders with a less clear allergic pathophysiology including primary eosinophil-associated GI disorders (EGID) (Hogan and Rothenberg, 2006), inflammatory bowel disease (IBD) (Lampinen et al., 2004; Jacobs et al., 2021) and DGBI (Salvo-Romero et al., 2020; Wauters et al., 2020). Very often, eosinophils accumulate together with mast cells in homeostasis, as well as under inflammatory conditions, besides sharing developmental and functional characteristics (Robida et al., 2018). In addition, physical interactions between eosinophils and mast cells have been demonstrated *in vitro* (Minai-Fleminger et al., 2010) as well as in asthma and nasal polyps (Elishmereni et al., 2011). Th2-cell released IL-4, -5 and -13 can activate mast cells in a similar fashion as eosinophils to secrete histamine, which in turn can activate eosinophils (Powell et al., 2010). Eosinophils are also thought to possess direct mast cell regulating capacities *via* their granular content since MBP stimulated mast cells to release mediators including histamine (Piliponsky et al., 2002). Moreover, incubation of rat peritoneal mast cells with MBP, EPO and ECP – but not EDN – also triggered histamine release (Piliponsky et al., 2002). Alternatively, locally produced corticotropin-releasing hormone (CRH) by eosinophils in the GI tract in response to substance P or acetylcholine can activate mast cells (Zheng et al., 2009; Wallon et al., 2011). Eosinophils themselves are also known to have neuromodulating properties by stimulating substance P release from dorsal root ganglion neurons, likely *via* MBP (Garland et al., 1997). Meanwhile, eosinophil migration can be triggered *via* a number of neuropeptides including substance P (Dunzendorfer et al., 1998). Lastly, eosinophils are actively recruited to nerves in a CCR3-dependent manner (Fryer et al., 2006).

These physical and functional interactions further substantiate the concept of an eosinophil-mast cell axis (Powell et al., 2010), which may explain the joint infiltration of eosinophils and mast cells in FD as discussed in the next paragraph. In addition, bidirectional neuro-immunological communication could provide an explanation for inflammation-induced neuronal hypersensitivity underlying symptom generation.

## Immune Activation And Eosinophil-Mast Cell Interactions In Functional Dyspepsia

### Cellular Signs of Immune Activation in Functional Dyspepsia

DGBI are classically regarded as functional disorders where no systemic, organic or metabolic cause can explain symptoms, as defined by the Rome IV criteria (Stanghellini et al., 2016). An initial report on mucosal eosinophilia in pediatric patients was the first indication for duodenal alterations in FD although no controls were included (Friesen et al., 2002). However, duodenal eosinophilia compared to healthy controls was later confirmed in an Australian pediatric cohort (Wauters et al., 2017). With the first evidence of increased duodenal eosinophil infiltration in adult FD patients, demonstrated in a Swedish population-based study (Talley et al., 2007), the duodenum became a hot topic in FD research. Importantly, in this cohort of ‘non-ulcer dyspepsia’ patients, eosinophil infiltration in the duodenal mucosa was associated with early satiety, a hallmark symptom of FD and especially PDS, suggesting a link between duodenal impairments and meal-related complaints. More studies have corroborated these initial findings of duodenal eosinophilia over the years (Walker et al., 2009, 2010, 2014; Tanaka et al., 2016; Sarkar et al., 2020; Wauters et al., 2021a), often confirming the link with FD symptoms, while no differences in mucosal eosinophil counts between FD and controls were found in other studies (Du et al., 2016; Järbrink-Sehgal et al., 2020; Nojkov et al., 2020; Puthanmadhom Narayanan et al., 2021) (Table 1). It was estimated that up to 40% of FD patients present with micro-inflammation, often eosinophil infiltration (Du et al., 2018).

**TABLE 1:** Evidence for immune activation in functional dyspepsia at the cellular level.

Findings	Associations	Methods	Population	Study details
**Duodenal mucosal immune activation**				
Eosinophil infiltration and degranulation		H&E, EM	20 pediatric FD (Rome II), uncontrolled	United States, 2002 (Friesen et al., 2002)
↑ Epithelial CD8^+^ CD3^+^ cell infiltration		Flow cytometry	6 *H. pylori*^+^ FD (Rome II) vs. 12 HC	France, 2007 (Gargala et al., 2007)
↑ Eosinophil infiltration	∼ early satiety	H&E	51 NUD (Rome II) vs. 48 HC	Sweden, 2007 (Talley et al., 2007)
↑ Eosinophil infiltration		H&E	51 NUD (Rome II) vs. 48 HC	Sweden, 2009 (Walker et al., 2009)
↑ Presence of CD8^+^ T cell aggregates	∼ gastric emptying	IHC	12 presumed pi FD vs. 12 unspecified FD	Belgium, 2009 (Kindt et al., 2009a)
↑ CD68^+^ cells				
↑ Histological duodenitis	∼ epigastric burning	H&E, IHC, IF	35 presumed pi FD (Rome III) vs. 20 HC	Japan, 2010 (Futagami et al., 2010)
↑ Eosinophil, CD68^+^ cell and CCR2^+^ macrophage infiltration				
↑ Eosinophil infiltration	∼ allergy	H&E	19 PDS (Rome III) vs. 89 HC	United Kingdom, 2010 (Walker et al., 2010)
↑ Eosinophil and mast cell infiltration	∼ TJ gene expression	IHC	15 FD (Rome III) vs. 15 HC	Belgium, 2014 (Vanheel et al., 2014)
↑ Eosinophil infiltration	∼ PDS symptoms & abdominal pain	H&E	33 FD (Rome II) vs. 22 HC	Australia, 2014 (Walker et al., 2014)
↑ Eosinophil and mast cell infiltration	∼ neuronal responses & PDS symptoms	IF (submucosa)	18 FD (Rome III) vs. 20 HC	Belgium, 2015 (Cirillo et al., 2015)
↑ Eosinophil and mast cell infiltration and degranulation		H&E, IHC, TB	141 FD (Rome III) vs. 39 HC	China, 2015 (Wang et al., 2015)
↑ Mast cell infiltration and degranulation		TB	48 FD (Rome III) vs. 21 HC	China, 2015 (Yuan et al., 2015a, b)
↑ Eosinophil degranulation		IHC	96 FD (Rome III) vs. 24 HC	China, 2016 (Du et al., 2016)
↑ Eosinophil infiltration		IF	9 FD (Rome III) vs. 5 HC	Japan, 2016 (Tanaka et al., 2016)
↑ Eosinophil infiltration	∼ weight loss	H&E	36 pediatric FD (Rome III) vs. 36 HC	Australia, 2017 (Wauters et al., 2017)
↑ Eosinophil and mast cell infiltration, eosinophil degranulation		IHC, EM	24 FD (Rome III) vs. 37 HC	Belgium, 2018 (Vanheel et al., 2018)
↑ Eosinophil infiltration and degranulation	∼ presence of fine nerve fibers	H&E, EM	51 FD (Rome III) vs. 35 HC	South Korea, 2019 (Lee et al., 2019)
↑ Eosinophil and mast cell infiltration		IHC	35 FD (Rome III) vs. 31 HC	Japan, 2019 (Taki et al., 2019)
Eosinophil degranulation	∼ FD symptoms	H&E	178 FD (Rome II) vs. 249 HC	United States, 2020 (Järbrink-Sehgal et al., 2020)
↑ Eosinophil infiltration	∼ FD & PDS symptoms	H&E	42 FD (Rome III) vs. 42 HC	Bangladesh, 2020 (Sarkar et al., 2020)
↑ Mast cell infiltration	∼ GI symptoms	IF (submucosa)	13 FD (Rome III) vs. 24 HC	Italy/Belgium, 2020 (Giancola et al., 2020)
↑ Eosinophil and mast cell infiltration	∼ FD symptoms	H&E, IHC	28 FD (Rome IV) vs. 30 HC	Belgium, 2021 (Wauters et al., 2021a)
**Gastric mucosal immune activation**				
↑ Histological gastritis		H&E	35 presumed pi FD (Rome III) vs. 20 HC	Japan, 2010 (Futagami et al., 2010)
↑ EC infiltration, mast cell and EC activation		IHC	30 presumed pi FD (Rome III) vs. 20 HC	China, 2010 (Li et al., 2010)
↓ CD206^+^ macrophage and ICC abundance		IHC (myenteric plexus)	9 FD (Rome III) vs. 9 HC	United States, 2021 (Pasricha et al., 2021)
**Systemic immune activation**				
↑ CD3^+^ CD45RO^+^ CD45RA^+^ cells		Flow cytometry	23 FD (Rome II) vs. 32 HC	Belgium, 2009 (Kindt et al., 2009b)
↑ CD4^+^ α_4_β_7_^+^ CCR9^+^ cells	∼ GI symptoms & gastric emptying	Flow cytometry	45 FD (Rome II) vs. 35 HC	Australia, 2011 (Liebregts et al., 2011)

*H&E: hematoxylin & eosin, EM: electron microscopy, FD: functional dyspepsia, NUD: non-ulcer dyspepsia, HC: healthy control, IHC: immunohistochemistry, pi: post-infectious, IF: immunofluorescence, PDS: postprandial distress syndrome, TJ: tight junction, TB: toluidine blue, GI: gastrointestinal, EC: enterochromaffin cells, ICC: interstitial cell of Cajal.*

**FIGURE 1:**
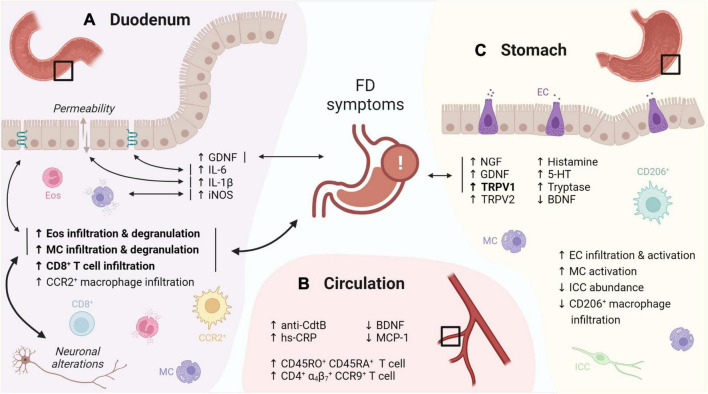
Graphical overview of the evidence for immune activation in FD. **(A)** The duodenal immune infiltrate in FD is characterized by increased eosinophil and MC infiltration and degranulation, linked to FD symptoms, neuronal alterations and changes in TJ gene expression. Increased levels of GDNF, IL-6, -1β and iNOS were linked to FD symptoms, TJ gene expression, increased permeability and mast cell degranulation, respectively. **(B)** Increased CD45RO^+^ CD45RA^+^ and gut-homing (α_4_β_7_^+^ CCR9^+^) lymphocytes were reported in the peripheral circulation of FD patients. In addition, increased anti-CdtB antibodies and hs-CRP, as well as decreased BDNF and MCP-1 were described. **(C)** Gastric immune alterations in FD include increased EC infiltration and activation, increased MC activation as well as decreased CD206^+^ macrophage and ICC abundance, although none were linked to symptoms. Increased gastric NGF, GDNF and TRPV1 levels in FD patients were associated to symptoms, along with increased TRPV2, histamine, 5-HT, tryptase and decreased BDNF. Confirmed findings are in bold. Double-headed arrows indicate associations, with confirmed associations in bold. FD: functional dyspepsia, MC: mast cell, TJ: tight junction, GDNF: glial cell-derived neurotrophic factor, iNOS: inducible nitric oxide synthase, CdtB: cytolethal distending toxin B, hs-CRP: high-sensitivity C-reactive protein, BDNF: brain-derived neurotrophic factor, MCP: monocyte chemoattractant protein, EC: enterochromaffin cells, ICC: interstitial cell of Cajal, NGF: nerve growth factor, TRPV: transient receptor potential vanilloid, 5-HT: serotonin. This figure was created in BioRender.

On top of increased infiltration with eosinophils, their activation and subsequent degranulation was frequently observed in later studies (Wang et al., 2015; Du et al., 2016; Vanheel et al., 2018; Lee et al., 2019). Despite similar leukocyte numbers, duodenal eosinophil degranulation was linked to FD symptoms in an ethnically diverse population (Järbrink-Sehgal et al., 2020), suggesting a role of duodenal eosinophils in symptom generation in FD. Moreover, duodenal immune activation was also linked with impaired mucosal integrity (Vanheel et al., 2014), and both neuronal and structural alterations (Cirillo et al., 2015; Lee et al., 2019), indicative for an integrated duodenal malfunctioning (Figure 1A).

Apart from eosinophils, mast cell infiltration and degranulation has also been reported (Vanheel et al., 2014, 2018; Cirillo et al., 2015; Wang et al., 2015; Yuan et al., 2015a,b; Taki et al., 2019; Giancola et al., 2020; Wauters et al., 2021a), although not always confirmed (Walker et al., 2009; Tanaka et al., 2016; Nojkov et al., 2020; Puthanmadhom Narayanan et al., 2021), supporting the concept of an eosinophil-mast-cell axis in FD (Powell et al., 2010). Increased infiltration of intraepithelial CD8^+^ lymphocytes, CCR2^+^ macrophages and CD68^+^ cells was found in post-infectious (pi) FD patients, implicating other inflammatory cell types in the observed immune activation (Gargala et al., 2007; Kindt et al., 2009a; Futagami et al., 2010). However, the inflammatory infiltrate in pi FD may be different from that of non-pi FD patients due to frequent overlap with pi IBS (Futagami et al., 2015). Indeed, besides mast cell activation, the gastric mucosa of pi FD patients also showed increased enterochromaffin cell (EC) infiltration and activation, accompanied by higher levels of released mediators including tryptase, histamine and serotonin (Li et al., 2010). In diarrhea-predominant IBS patients, duodenal intraepithelial lymphocyte (IEL) and mast cell numbers were higher compared to healthy controls, which was linked to impaired serotonin uptake by platelets (Foley et al., 2011). Various studies also implicated serotonin metabolism in other IBS subtypes (Holtmann et al., 2017), but not FD. However, increased EC number and mediator release in pi FD could result in altered serotonin metabolism (Berger et al., 2009). Of interest, serotonin is a potent eosinophilic chemoattractant, suggested to complement eotaxin-mediated chemotaxis (Boehme et al., 2004). Besides altered numbers, more EC and activated mast cells were present near nerve fibers (< 5μm) in the stomach of FD compared to healthy controls (Li et al., 2010). These changes in tissue organization were later corroborated as more submucosal eosinophils and mast cells localized close to nerves in the duodenum of FD patients compared to healthy controls (Cirillo et al., 2015; Giancola et al., 2020). The presence of duodenal fine nerve fibers also strongly correlated with increased eosinophil infiltration in FD (Lee et al., 2019).

Systemic changes, including increased CD45RA and CD45RO double positive cells, likely representing naive T cells in transition to a memory phenotype, were reported in FD (Kindt et al., 2009b). Of interest, another study described increased circulating small-intestinal homing (α_4_β_7_^+^ CCR9^+^) lymphocytes which were associated to various GI symptoms and gastric emptying (GE) (Liebregts et al., 2011) (Figure 1B). This suggests that increased lymphocyte recruitment to the small bowel, indicative of an active inflammatory state, may lead to gastric dysfunction and thus contributes to FD pathophysiology, although confirmation is needed.

Remarkably, only few studies reported alterations in the immune cell profile of the stomach and none were related to FD symptoms (Futagami et al., 2010; Li et al., 2010; Pasricha et al., 2021), thereby confirming the poor link between symptoms and gastric pathophysiology (Figure 1C). This supports the concept of the duodenum as primary pathogenic center (Wauters et al., 2020) with impaired duodenogastric communication contributing to the typical FD symptomatology (Lee and Tack, 2010; Vanheel and Farre, 2013; Wauters et al., 2021b).

Overall, a recent systematic review and meta-analysis by Du and colleagues confirmed that eosinophil [standard mean difference (SMD) = 0.36, *p* = 0.03] and mast cell (SMD = 0.94, *p* = 0.007) counts in the stomach were indeed increased in FD patients compared to controls, with even more convincing evidence for duodenal eosinophil (SMD = 0.95, *p* < 0.001) and mast cell (SMD = 0.66, *p* = 0.005) infiltration, but not for other cell types (Du et al., 2018). Conflicting histological findings among studies could be attributed to variation in Rome definitions used for patient inclusion (ranging from Rome II to the latest Rome IV consensus in the studies discussed in this Review), while also the lack of a clear cut-off and counting procedures hampers uniform assessment of eosinophil infiltration and thus limits generalizability.

### Molecular Signs of Immune Activation in Functional Dyspepsia

Besides increased mucosal infiltration of inflammatory cells and subsequent release of their granular components, altered presence of soluble factors in the mucosal environment further substantiates the evidence for immune activation in FD (Table 2). A recent Japanese study found that increased duodenal gene expression of IL-1β was associated to decreased mucosal impedance indicating increased epithelial permeability, which in turn was linked to decreased expression of zonula occludens-1 (ZO-1), a key protein of the intercellular tight junction (Komori et al., 2019). Similarly, Nojkov et al. reported an inverse association between increased expression of IL-6, and claudin-2 and -4 (Nojkov et al., 2020). Hence, both studies strengthened the link between an impaired duodenal barrier and immune activation as described earlier (Vanheel et al., 2014). However, whether immune dysregulation precedes the barrier defect or *vice versa* remains to be determined. These two pathophysiological events most likely amplify each other, leading to a vicious circle, which complicates untangling these mechanisms even more.

**TABLE 2:** Evidence for immune activation in functional dyspepsia at the molecular level.

Findings	Associations	Methods	Population	Study details
**Duodenal mucosal immune activation**				
↑ iNOS	∼ mast cell degranulation and abdominal distention	IHC	48 FD (Rome III) vs. 21 HC	China, 2015 (Yuan et al., 2015a)
↑ GDNF	∼ epigastric burning	ELISA	9 FD (Rome III) vs. 5 HC	Japan, 2016 (Tanaka et al., 2016)
↑ IL-1β	∼↓ duodenal mucosal impedance (↑permeability)	qPCR	24 FD (Rome III) vs. 20 HC	Japan, 2019 (Komori et al., 2019)
↑ IL-6	∼ TJ gene expression	qPCR	18 FD (Rome IV) vs. 20 HC	United States, 2020 (Nojkov et al., 2020)
**Gastric mucosal immune activation**				
↑ Histamine, ↑ 5-HT and ↑ tryptase		ELISA, HPLC, western blot	30 presumed pi FD (Rome III) vs. 20 HC	China, 2010 (Li et al., 2010)
↑ NGF, ↑ GDNF and ↑ TRPV1	∼ epigastric pain/burning and postprandial fullness	qPCR, IHC	117 FD (Rome III) vs. 55 HC	South Korea, 2016 (Choi et al., 2016)
↓ BDNF, ↑ TRPV1 and -2		qPCR	36 PDS (Rome IV) vs. 23 HC	China, 2018 (Cheung et al., 2018)
IL-6	∼ epigastric burning			
**Systemic immune activation**				
↑ IL-5, ↑ IL-13 and ↓ IL-10 (stimulated)		ELISA (stimulated PBMC culture supernatants)	23 FD (Rome II) vs. 32 HC	Belgium, 2009 (Kindt et al., 2009b)
↓ IL-12 (stimulated)		ELISA (stimulated monocyte culture supernatants)	23 FD (Rome II) vs. 32 HC	Belgium, 2009 (Kindt et al., 2009b)
↑ TNF-α, ↑ IL-1β and ↑ IL-10	∼ GI symptoms and gastric emptying	ELISA (PBMC culture supernatants)	45 FD (Rome II) vs. 35 HC	Australia, 2011 (Liebregts et al., 2011)
↓ BDNF and ↓ MCP-1		ELISA and multiplex	36 PDS (Rome IV) vs. 23 HC	China, 2018 (Cheung et al., 2018)
Peripheral cytokines	∼ FD and GI symptoms			
↑ anti-CdtB antibodies		ELISA	61 FD (Rome III) vs. 245 HC	Australia, 2019 (Talley et al., 2019)
↑ hs-CRP		Turbidimetry	28 FD (Rome IV) vs. 30 HC	Belgium, 2021 (Wauters et al., 2021a)

*iNOS: inducible nitric oxide synthase, IHC: immunohistochemistry, FD: functional dyspepsia, HC: healthy control, GDNF: glial cell-derived neurotrophic factor, ELISA: enzyme-linked immunosorbent assay, IL: interleukin, qPCR: quantitative polymerase chain reaction, 5-HT: serotonin, HPLC: high-performance liquid chromatography, pi: post-infectious, NGF: nerve growth factor, TRPV: transient receptor potential vanilloid, BDNF: brain-derived neurotrophic factor, PDS: postprandial distress syndrome, PBMC: peripheral blood mononuclear cells, TNF: tumor necrosis factor, GI: gastrointestinal, MCP: monocyte chemoattractant protein, CdtB: cytolethal distending toxin B, hs-CRP: high-sensitivity C-reactive protein.*

Evidence for neuro-immunological mechanisms on the molecular level include increased inducible nitric oxide synthase (iNOS) in the duodenum, which was linked to mast cell degranulation as well as upper abdominal distention scores in PDS patients (Yuan et al., 2015a). In addition, increased glial cell line-derived neurotrophic factor (GDNF) levels, a neurotrophin also produced by eosinophils, correlated with the intensity of epigastric burning in FD (Tanaka et al., 2016). Similar findings were described in the gastric mucosa where GDNF and nerve growth factor (NGF) were increased in FD patients, with associations to epigastric pain or burning, postprandial fulness, but not early satiety (Choi et al., 2016). Brain-derived neurotrophic factor (BDNF), another neural growth factor expressed in the intestine, was decreased in the gastric mucosa as well as in the circulation of FD patients (Cheung et al., 2018). Interestingly, mice with reduced intestinal BDNF expression showed increased vagal sensory innervation with restricted feeding and possibly associated increased satiation (Biddinger and Fox, 2014). Several studies already proposed a role for BDNF in IBS and abdominal pain (Yu et al., 2012; Konturek et al., 2020). Moreover, interactions between eosinophils and neurons through neurotrophic factors including BDNF but also NGF (Raap and Wardlaw, 2008), both produced by eosinophils (Noga et al., 2003), could provide a pathophysiological mechanism to understand the emergence of GI symptoms following immune activation as observed in FD and other DGBI. Likewise, key eosinophil proteins MBP and EPO act as antagonists of M2 muscarinic receptors, potentially resulting in receptor dysfunction and vagally increased smooth muscle reactivity that could underly disturbed GI motility (Jacoby et al., 1993; Walker et al., 2010).

Interestingly, release of peripheral cytokines including typical Th2 cytokines IL-4 and -5, was significantly associated to dyspeptic symptoms in FD (Cheung et al., 2018). Cheung et al. (2018) also reported increased transient receptor potential vanilloid (TRPV)1 and -2 expression in the stomach. The role of TRPV1 in DGBI is most established for IBS where histamine-1 receptor (H_1_R)-mediated sensitization of TRPV1 contributed to the development of visceral hypersensitivity (Wouters et al., 2016). In a placebo-controlled trial, ketotifen relieved abdominal pain and increased the threshold for discomfort in hypersensitive IBS patients, although it remained unclear whether mast-cell stabilization or H_1_R antagonism was responsible (Klooker et al., 2010). More recently, a 12-week treatment with the H_1_R antagonist ebastine decreased abdominal pain and reduced hypersensitivity to distention compared to placebo, with inhibition of H_1_R-mediated neuronal sensitization rather than immunomodulatory properties suggested to underly this effect (Wouters et al., 2016). Also, TRPV1 and -3 were implicated in chemosensitivity to lipids in IBS patients, potentially resulting in postprandial symptoms, while increased duodenal TRPV1 expression was linked to abdominal and rectal pain scores (Grover et al., 2021). Of note, hypersensitivity to oral intake of capsaicin – a major TRPV1 agonist – has been demonstrated in FD patients (Hammer, 2018), which could be explained by increased TRPV1 expression. In addition, exogenous palmitoylethanolamide (PEA), a TRPV1 agonist, counteracted increased mast cell infiltration and TRPV1 and -4 upregulation after acid exposure in FD, while the normal acid-induced secretion of endogenous PEA was suppressed in FD versus control biopsies (Sarnelli et al., 2021).

## Factors Related To Immune Activation

### Gut-Brain Axis

**FIGURE 2:**
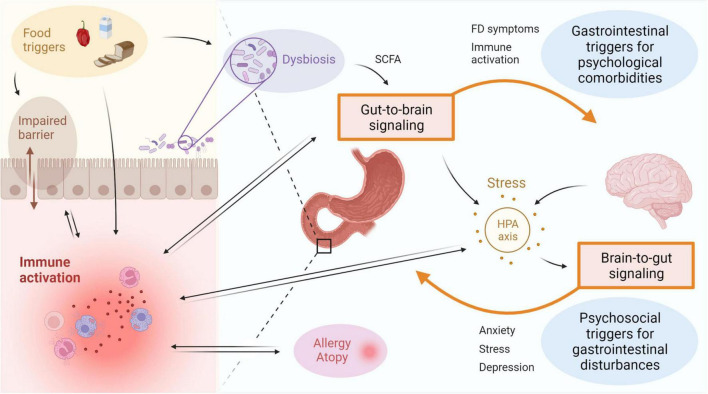
Immune activation is linked to disturbances of the gut-brain-axis in FD. Bidirectional interaction between the gut and the brain involves GI symptoms and immune activation as potential triggers for psychological comorbidities, as well as psychosocial factors including stress, anxiety or depression resulting in gastroduodenal alterations. Activation of the HPA axis is suggested to play an important role, with both central and gut-derived mediators directly leading to induction of stress, which in turn can trigger GI symptoms. Factors associated to the intestinal inflammatory environment include microbial alterations and barrier impairments, both of which can be a cause or consequence of immune activation. The gut microbiota is also suggested to influence gut-to-brain communication directly or indirectly *via* metabolites such as SCFA. Food triggers are a major determinant of the microbiota composition and can also directly induce immune activation and impact barrier function. A relation between duodenal immune activation and allergy or atopy is proposed, but directionality and causality need to be established. FD: functional dyspepsia, GI: gastrointestinal, HPA: hypothalamic-pituitary-adrenal, SCFA: short-chain fatty acids. This figure was created in BioRender.

Multiple factors have been proposed to be associated with duodenal immune activation in FD patients (Figure 2). An important bidirectional interaction exists between the intestine and the brain, also known as the gut-brain-axis (GBA), and this is thought to be a major contributor to the emergence of FD symptoms (Wauters et al., 2021c). Psychosocial factors including anxiety and mood disorders were shown to be independently associated with the onset of FD and PDS, indicative of brain-gut signaling (Aro et al., 2009; Jones et al., 2017). Alternatively, in a subset of DGBI patients, a primary gut-driven syndrome precedes the development of brain-related symptoms in a gut-to-brain mechanism, thereby confirming bidirectionality of the gut-brain interaction (Koloski et al., 2016). The proportion of DGBI patients with primary brain-to-gut signaling was estimated at 1/3, whereas gut-to-brain signaling accounted for 2/3 of the cases (Koloski et al., 2016).

As an example, both duodenal mast cell counts and degranulation rate correlated with anxiety and depression symptoms in FD patients (Yuan et al., 2015b). The concept of gut-brain signaling was also supported by an increased odds of developing anxiety for baseline duodenal eosinophilia after 10-year follow-up in a Swedish population-based cohort (Ronkainen et al., 2021). Although a link between duodenal eosinophils and anxiety independent of FD was suggested, the effect size was not significant when controlling for baseline FD status (Ronkainen et al., 2021). Large prospective studies should address the mechanisms by which psychobiological factors relate to DGBI since the limited number of patients with duodenal eosinophilia presenting with anxiety hampered firm conclusions on the interplay between FD status, duodenal eosinophilia and anxiety (Wauters et al., 2021c). Moreover, the use of questionnaires assessing depression, anxiety or other psychological comorbidities should be complemented by biochemical analyses on the level of the hypothalamic-pituitary-adrenal (HPA) axis as discussed below (Ronkainen et al., 2021).

### Stress

CRH is an important effector in the HPA axis, typically increased in a central stress response. However, it can also be produced and secreted locally by intestinal eosinophils in response to psychological stress (Zheng et al., 2009; Wallon et al., 2011). Wild-type rats experienced colonic barrier dysfunction after chronic peripheral CRH administration, while mast cell-deficient animals did not, implying a mast cell-mediated mechanism of barrier impairment (Teitelbaum et al., 2008). Indeed, the involvement of mast cells in the emergence of a barrier defect following peripheral CRH administration was corroborated in healthy controls since it could be blocked by pretreatment with cromoglycate, a mast cell stabilizer, while a strikingly similar effect was observed in response to acute psychological stress (Vanuytsel et al., 2014). Functional dyspepsia patients also reported higher stress levels accompanied by increased fullness, a critical FD symptom, compared to healthy controls, and both were positively associated with one another in patients but not controls (Klaassen et al., 2021). This suggests a direct relationship between stress and FD symptoms, in which increased duodenal eosinophil and mast cell numbers may play a role. This concept is supported by our recent findings of increased salivary awakening cortisol levels as a marker of stress in FD patients, with a decrease after 4-week proton pump inhibitor (PPI) therapy compared to baseline (Wauters et al., 2021a). Moreover, reduction in eosinophils on-PPI (see section “Therapeutic options targeting immune activation in FD”) was associated with changes in cortisol, highlighting the impact of duodenal immune activation in FD (Wauters et al., 2021a).

Classical pro-inflammatory cytokines including IL-6, tumor necrosis factor (TNF)-α and IL-1β act as potent activators of the HPA axis, confirming the relation between stress and immune activation. These cytokines can exert stimulatory effects on CRH release in the brain, thereby inducing a stress response (Powell et al., 2017). In an IBS rat model of maternal separation stress, CRH stimulated colonic IL-6 secretion, thereby affecting neuro-secretory functions (O’Malley et al., 2013). In addition, another study using maternal separation as a stress model in mice found that the stress-induced increase in IL-6, TNF- α and iNOS and increased colonic permeability was inhibited by selective blocking of CRH receptor (CRHR)1 but not CRHR2 (Li et al., 2017). This confirms the previously proposed pro-inflammatory role of CRHR1 as opposed to mucosal barrier restorative capacities of CRHR2 (Im et al., 2010). As IL-6, TNF-α and IL-1β have all been shown to be upregulated in the duodenal mucosa (Komori et al., 2019; Nojkov et al., 2020) or peripheral blood mononuclear cell (PBMC) supernatants from FD patients (Liebregts et al., 2011), they might also be involved in the reciprocal relationship between stress and FD pathophysiology.

### Allergy, Atopy and Adverse Food Reactions

As eosinophils and mast cells are classically involved in allergic conditions, allergy and atopy are indeed associated with DGBI. Duodenal eosinophilia was common in allergic patients {odds ratio (OR) [95% confidence interval (CI)] 5.04 [2.12–11.95]} without an association to specific upper GI symptoms (Walker et al., 2010). Asthma was more prevalent in patients with FD (OR 1.41 [1.26–1.58]), which was even more the case in patients with multiple DGBI (Jones et al., 2014). A later study corroborated these findings as asthma (OR 1.32 [1.04–1.68]) and food allergy (OR 1.78 [1.28–2.49]) were found to be independent predictors for FD, controlling for potential confounders such as age, sex and psychological distress (Koloski et al., 2019). Similarly, lower GI symptoms were more common in patients with asthma (OR 2.13 [1.39–2.56]) and allergic rhinitis (OR 1.47 [1.04–2.10]) compared to the non-allergic population (Powell et al., 2007). Nevertheless, no causal relationship between allergy, atopy and DGBI, nor directionality of these associations have been described so far.

In spite of multiple studies linking allergies to DGBI, these patients do not experience more IgE-mediated food responses compared to the general population. The prevalence of food hypersensitivity in DGBI patients was estimated to be 4% (8/200) (Ismail et al., 2018), which is in line with overall prevalence numbers ranging from 1 to 4% (Skypala, 2011). However, 9.5% (19/200) of the DGBI patients tested positive for specific serum IgE against 6 common food allergens, highlighting the imperfect link between serum IgE and clinical symptoms (Ismail et al., 2018). In contrast, food antigen specific IgG antibodies in serum of FD patients were significantly higher than in controls, but not linked to symptom severity (Zuo et al., 2007). More recently, the attention in DGBI research also shifted to non-IgE mediated adverse food reactions. A German study using duodenal confocal laser endomicroscopy (CLE) in IBS patients found that more than half (76/108) responded to CLE food challenge despite having negative IgE serology (Fritscher-Ravens et al., 2019). Moreover, atopy was 4-times more prevalent in responders compared to controls and immediate immune activation was evident from increased eosinophil degranulation and luminally released ECP (Fritscher-Ravens et al., 2019). Also, in an “immuno-active” group of IBS patients, genes coding for the pro-inflammatory cytokine IL-1β and mast cell G-protein coupled receptor MRGPRX2, as well as the cyclooxygenase gene PTGS2 were significantly upregulated in the colon compared to healthy controls (Aguilera-Lizarraga et al., 2019). All of these have been implicated in mast cell activation (Aguilera-Lizarraga et al., 2019), with MRGPRX2 in specific mediating IgE-independent mast cell activation in response to various ligands including eosinophil cationic proteins (Ali, 2016; Kumar et al., 2021).

Besides, the relatively new clinical entity of non-celiac gluten sensitivity (NCGS), defined as both GI and extra-intestinal reactions in response to gluten intake in the absence of wheat allergy and celiac disease, shares a highly similar symptom pattern to DGBI (Carroccio et al., 2012; Catassi et al., 2015; Barbaro et al., 2020). Indeed, a recent study found that 35% (27/77) of refractory FD patients responded to a gluten-free diet and in 18% (5/27) of these, symptoms recurred after gluten-rechallenge, suggesting NCGS (Shahbazkhani et al., 2020). However, controversy still remains on the topic as a study reported that fructans, rather than gluten, triggered symptoms in NCGS (Skodje et al., 2018). A recent pilot study reported slight symptom improvement in FD patients after a gluten-free and low fermentable oligo-, di-, mono-saccharides, and polyols (FODMAP) diet, but without identification of a specific causal component (Potter et al., 2020a).

A study in IBS found that 84% of 197 included patients experienced symptoms after intake of specific foods, and multiple food triggers were linked to more severe symptoms and lower quality of life (Böhn et al., 2013). Indeed, also allergy-independent mechanisms can provoke bothersome reactions as, e.g., spicy food intake was linked to fullness and retching symptoms in FD patients, irrespective of TRPV1 genotype (Lee et al., 2016). As a consequence, the burden imposed by food-related reactions can be enormous with an alarming rate of 39.8% (37/93) of DGBI patients reporting symptoms of avoidant or restrictive food intake disorders (ARFID), which could be triggered or worsened by dietary restrictions as part of (self-initiated) therapy (Burton Murray et al., 2021).

Taken together, atopy, allergies and untoward food reactions are linked to various DGBI although more mechanistic studies are needed to untangle the immunological maze of factors contributing to overlap between these conditions, and mechanisms primarily driving GI symptom generation, respectively. In addition, studies focusing on the immune-related pathophysiology in DGBI should acknowledge the presence of atopy and allergy as potential confounders in order to isolate the drivers of DGBI symptoms (Wauters et al., 2021d). Only by doing so, the way is paved for targeted therapies to be developed.

### Impaired Barrier Function

The intestinal barrier physically separates the lumen from the surrounding gut wall, maintaining an equilibrium between nutrient uptake and defending the body against harmful intruders. Upon disturbance of this homeostatic relationship, luminal contents may invade the intestinal tissue, as is proposed in DGBI (Wauters et al., 2019). This increased permeability was first reported in pi IBS, along with increased rectal lymphocyte counts (Spiller et al., 2000). With the first description of duodenal hyperpermeability in FD by our group, a potential novel pathophysiological mechanism was identified (Vanheel et al., 2014). Mucosal integrity, measured in Ussing chambers, was impaired in FD versus healthy controls, and downregulated expression of barrier-related genes was associated to increased permeability as well as infiltration of eosinophils and mast cells (Vanheel et al., 2014). More recently, these *ex vivo* findings were corroborated *in vivo* using CLE (Nojkov et al., 2020) and mucosal impedance measurements (Ishigami et al., 2017).

Although an impaired barrier and immune activation have been repeatedly linked to one another, determining the causal link proved to be extremely difficult. Barrier impairment could be secondary to multiple triggers including luminal substances or factors released by the inflammatory infiltrate. Duodenal acid perfusion resulted in increased permeability in healthy volunteers, while increased tryptase expression suggested mast cell activation. However, pretreatment with the mast cell stabilizer cromoglycate did not prevent barrier impairment, pointing toward direct acid-related effects instead of a mast-cell dependent mechanism (Vanheel et al., 2020). Nevertheless, mast cells are known regulators of permeability (Wallon et al., 2008) and eosinophil-activated mast cells can cause barrier dysfunction, whereas activated eosinophils alone did not alter epithelial permeability (Wallon et al., 2011). In contrast, utilizing the same T84 epithelial cell line in another study, eosinophils were found to increase permeability, mediated by MBP (Furuta et al., 2005). We recently confirmed that *ex vivo* permeability in Ussing chambers was higher in FD patients compared to controls, and that a 4-week course of PPI restored barrier function to levels of healthy controls (Wauters et al., 2021a). This effect was, however, not directly related to anti-inflammatory effects of PPI (Wauters et al., 2021a), suggesting that other mechanisms, potentially *via* luminal agents, may contribute.

### Microbial Factors

Although physically separated by the intestinal barrier, luminal contents are major determinants of mucosal homeostasis and immune activation, with the gut microbiome being of considerable importance (Peterson and Artis, 2014). Increased presence of luminal *Streptococcus* was reported in FD compared to controls and correlated to upper GI symptoms in both PDS and EPS subgroups (Fukui et al., 2020). We recently provided evidence for the involvement of luminal *Porphyromonas* in FD, with an inverse association of the genus abundance to GI symptoms and duodenal eosinophilia, suggesting a link with immune activation (Wauters et al., 2021g). In addition, the efficacy of a combination of two spore-forming probiotic *Bacillus* strains was demonstrated in a placebo-controlled study in FD, with decreased peripheral Th17 signaling and increased *Faecalibacterium* and *Roseburia* in stools, suggesting immunomodulatory properties of probiotic therapy in FD (Wauters et al., 2021f). In IBS, microbial alterations have been repeatedly demonstrated, with changes in mucosal and fecal bacterial composition, suggestive of small intestinal bacterial overgrowth (Giamarellos-Bourboulis et al., 2015; Li et al., 2018; Yang et al., 2020). Colonic microbial alterations in IBS were also linked to symptoms and immune activation (Sundin et al., 2020).

Besides compositional imbalance, metabolomic dysregulation could be of importance as well. Beneficial effects of short-chain fatty acids (SCFA) on intestinal inflammation were suggested by oral butyrate administration resulting in reduced colonic infiltration of neutrophils and eosinophils in mice during colitis, but not in healthy mice (Vieira et al., 2012). These bacterial metabolites are thought to be important mediators in gut-brain communication (Dalile et al., 2019), but further research on the role of SCFA and other microbial products in DGBI is needed. Overall, microbial alterations have been implicated in DGBI pathogenesis, even though a definitive dysbiotic signature relating to symptomatology is lacking. Further progress in the field of microbial research is eagerly awaited, especially on microbial induced changes in the mucosal immune profile in DGBI.

## Overlapping Disorders Of Gut-Brain Interaction

Frequently overlapping symptom patterns with other DGBI complicate the diagnosis and management of FD. FD and IBS are the most prevalent DGBI (Sperber et al., 2021a) and overlap between these disorders is common in clinical practice (von Wulffen et al., 2019). In a large proportion of patients, overlapping DGBI were present and resulted in an even worse disease severity and quality of life (Aziz et al., 2018; Sperber et al., 2021b). With an increasing number of complaints due to overlapping syndromes and comorbidities, also somatic symptom reporting may influence gastrointestinal symptom perception, which is thought to occur in both FD and IBS (Van Oudenhove et al., 2008, 2016; Heidari et al., 2017).

Whereas FD presents with symptoms thought to originate from the gastroduodenal region, IBS presents with lower GI complaints such as constipation, diarrhea and abdominal pain. Moreover, the inflammatory infiltrate in IBS predominantly consists of mast cells, initially reported in the terminal ileum (Weston et al., 1993) and cecum (O’Sullivan et al., 2000), but later also in other parts of the colon and even more proximally in the small intestine (Bashashati et al., 2018; Robles et al., 2019). However, the increased infiltration and degranulation of eosinophils in the colonic mucosa also suggested a role for eosinophils in IBS (Katinios et al., 2020; Salvo-Romero et al., 2020; Casado-Bedmar et al., 2021). To which extent this contributes to the frequent overlap between the two disorders is not yet understood.

A disorder highly similar to FD is gastroparesis, presenting with postprandial symptoms as seen in FD, but also with nausea and vomiting that are not regarded as typical FD symptoms (Stanghellini and Tack, 2014). The presence of delayed GE in the absence of a mechanical obstruction is a key diagnostic criterion in gastroparesis, and results most likely from neuromuscular dysfunction (Camilleri et al., 2018). Depending on the method used, optimally measured GE was associated with various cardinal gastroparesis symptoms (Vijayvargiya et al., 2019). Nevertheless, delayed GE appeared to be an unstable event over time as a recent study found that 42% (79/189) of patients initially diagnosed with gastroparesis were reclassified having FD after 48 weeks, whereas 37% (22/60) of baseline FD patients made the opposite diagnostic switch (Pasricha et al., 2021). The neuromuscular component in gastroparesis is well appreciated compared to other DGBI, with impaired interstitial cell of Cajal (ICC) structure associated with decreased anti-inflammatory CD206^+^ macrophage infiltration in the myenteric plexus of the gastric wall (Bernard et al., 2014; Grover et al., 2017). Intriguingly, both findings were also demonstrated in a small subset of FD patients compared to healthy controls, adding up to the overlap or even interchangeability of both disorders (Pasricha et al., 2021). Therefore, FD and gastroparesis are proposed to be regarded as related disorders at different ends of the intestinal neuromuscular disorder spectrum (Pasricha et al., 2021; Tack et al., 2021).

DGBI also exhibit frequent overlap with other non-DGBI including gastroesophageal reflux disease (GERD) (de Bortoli et al., 2018) or non-inflammatory connective tissue disorders including joint hypermobility syndrome and Ehlers-Danlos syndrome (Fikree et al., 2015; Carbone et al., 2020; Lam et al., 2021). Although the mechanisms explaining overlap are often unclear, visceral hypersensitivity, neuromuscular disturbances and reporting of multiple somatic symptoms could play a role (Fikree et al., 2015; de Bortoli et al., 2018; Carbone et al., 2020; Lam et al., 2021).

A better understanding of cellular and molecular changes underlying the pathophysiology in different DGBI is essential to fulfill the diagnostic and therapeutic unmet needs in the field. Eventually, implementing objective measures, of which the intestinal immune activation profile appears to be a prime candidate, in addition to symptomatologic evaluation as part of the diagnostic work-up could promote faster and more accurate diagnosis, and allow targeted therapies to be developed.

## Therapeutic Options Targeting Immune Activation In Functional Dyspepsia

Treatment options in FD are limited due to poor efficacy and safety concerns, with the exception of eradication therapy for *Helicobacter pylori*-positive patients (Moayyedi et al., 2017; Ford et al., 2022). PPI are still the first-line treatment option as recommended by North-American and European consensus guidelines (Moayyedi et al., 2017; Wauters et al., 2021e). However, acid suppression with PPI only showed a moderate increase in efficacy compared to placebo (risk ratio [95% CI] 0.88 [0.82–0.94]) and a number needed to treat of 11 in a recent Cochrane meta-analysis (Pinto-Sanchez et al., 2017). In addition, PPI therapy was associated with an increased risk for enteric infections (Freedberg et al., 2017; Moayyedi et al., 2019) and alterations of the gut microbiota composition toward a more oral-like flora (Imhann et al., 2016; Jackson et al., 2016), further discouraging the long-term use of these drugs in FD in case of insufficient clinical benefit. Moreover, until recently, it was poorly understood how acid-suppressive drugs improve dyspeptic symptoms in the absence of significant gastro-esophageal reflux.

Besides PPI, also histamine-2 receptor (H_2_R) antagonists suppress gastric acid secretion, albeit less powerful but with similar efficacy compared to PPI (Moayyedi et al., 2017; Tack et al., 2019). H_1_R antagonists are less frequently used and are considered anti-emetics. In IBS, however, H_1_R blockade decreased symptoms and visceral hypersensitivity compared to placebo while mast cell numbers remained unaffected (Wouters et al., 2016). Dual H_1_R and H_2_R inhibition was retrospectively evaluated in FD and resulted in symptom improvement, but without anti-inflammatory effects (Potter et al., 2020b). Prokinetic drugs increase gastric motility, thereby reducing intestinal transit time, suggesting their efficacy lies in patients with delayed gastric emptying (Lacy et al., 2012). The group of prokinetics is heterogeneous and includes 5-HT_4_-receptor agonists, dopamine-2 receptor antagonists and drugs increasing cholinergic effects, although their overall efficacy in FD remains unclear (Pittayanon et al., 2019).

The recognition of immune activation in FD has not yet led to major breakthroughs with specific anti-inflammatory drugs tackling the eosinophil infiltration. The leukotriene antagonist montelukast showed clinical benefit in a pediatric FD population presenting with increased eosinophil infiltration, although this was not related to a non-significant decrease in duodenal eosinophils (Friesen et al., 2009). An Australian case-control study reported decreased duodenal eosinophil counts in 10 FD patients on-PPI versus 10 untreated patients, suggesting an anti-eosinophilic effect of PPI (Potter et al., 2019). In addition, we recently provided the first prospective evidence for anti-inflammatory effects of PPI in the duodenum of FD patients (Wauters et al., 2021a). Twenty-eight PPI-naïve FD patients had higher duodenal eosinophil and mast cell numbers compared to 30 age- and sex matched healthy controls, which were significantly reduced in patients after a 4-week routine course of PPI (pantoprazole 40 mg once daily). Moreover, we demonstrated that those patients with the greatest reduction in mucosal eosinophils also reported the largest decrease in symptoms, indicating that anti-eosinophilic effects of PPI contributed to the therapeutic efficacy of these widely used drugs. Interestingly, this effect was independent of the acid-suppressant properties of PPI since it was not mediated by changes in duodenal pH, suggesting a direct effect on the inflammatory environment.

Anti-inflammatory effects of PPI have been reported before in other conditions. Non-gastric ATPases have been proposed to be involved in the PPI-mediated decrease in eotaxin-3 expression in chronic rhinosinusitis patients with nasal polyps (Min et al., 2017). Similarly, esophageal squamous cells derived from eosinophilic esophagitis (EoE) patients expressed non-gastric ATPases, which could be inhibited by L-type calcium channel inhibitors and potentially PPI (Odiase et al., 2021). Proton pump inhibitor also inhibited eotaxin-3 expression in EoE esophageal cells by blocking the binding of STAT6 to the eotaxin-3 promoter (Zhang et al., 2012; Cheng et al., 2013), providing additional evidence for an acid-independent mechanism of PPI, which could also be at play in the duodenum of PPI-treated FD patients. Janus kinase (JAK)-STAT6 pathway inhibitors that block eostaxin-3 expression were therefore suggested as a promising new therapeutic option in EoE (Cheng et al., 2016), but should also be explored in the context of FD. These proposed effects of PPI warrant further investigation to assess their potential in resolving eosinophil infiltration in the duodenum and subsequently confirm a similar mechanism of action in FD. Nevertheless, the capability of PPI to decrease eosinophilic inflammation in an acid-independent manner, thereby leading to symptom improvement, means considerable progress in recognizing eosinophils as important contributors to FD pathophysiology. Also, studies need to consider PPI-use among DGBI patient populations as a major confounder when looking at duodenal alterations (Wauters et al., 2021d), which is still often neglected to date.

An effort to assess specific anti-inflammatory therapies for FD was recently made by Talley et al. in a randomized placebo-controlled trial using budesonide, a locally acting corticosteroid with known efficacy in EoE (Talley et al., 2021). Even if there was no significant difference between both treatment groups, the authors did find a significant association between decreased duodenal eosinophil counts and a reduction of both postprandial fullness and early satiety, from baseline to post-treatment (Talley et al., 2021). Although this proof-of-concept study only included 11 FD patients, it confirms the link between duodenal immune activation and gastroduodenal symptoms, despite a lack of evidence supporting clinical efficacy of budesonide in FD.

The importance of eosinophilic infiltration in the pathogenesis of gastroduodenal disorders was also affirmed by the results of a novel eosinophil depleting and mast cell inhibiting anti-siglec-8 monoclonal antibody in eosinophilic gastritis and duodenitis (Dellon et al., 2020). Dellon et al. found significantly decreased eosinophil counts with two different dosages compared to placebo, which was accompanied by substantial symptom improvement. However, since this trial included eosinophilic gastritis and duodenitis patients with non-FD specific GI symptoms such as nausea and diarrhea, as well as significant duodenal eosinophil infiltration [defined as 30/high-power field (HPF) in at least 3 HPF], caution should be warranted when translating these findings to FD. Nevertheless, the potential benefit of these and other anti-inflammatory therapies for FD should be explored in future studies involving well-characterized patient cohorts. Accordingly, the latest Rome diagnostic criteria should be adopted for patient inclusion, as variability in symptom and subgroup characterization could skew the results of interventional trials.

## Future Perspectives

With immune activation increasingly becoming a hot topic in DGBI research, the accurate quantification of immune cell infiltration — where routine histology still remains the golden standard — is of utmost importance. The infiltration of eosinophils can be patchy in nature (Talley et al., 2007; Saffari et al., 2012), which could confound duodenal cell counts *via* conventional histology. Therefore, appropriate counting methods are essential for studies assessing low-grade inflammation (Walker et al., 2010). Also, caution is needed when interpreting spatial differences of eosinophil infiltration in the duodenal bulb (D1) versus the second portion of the duodenum (D2), as the implications of these differences remain unclear. A first step to shape clarity in the heterogeneous field of eosinophil counts is to uniformly express cell numbers per mm^2^ instead of per HPF, with the latter being dependent on the objective lens and the ocular of the microscope (Kiss et al., 2018). Furthermore, even though single-biopsy histology is an accessible technique suitable for routine diagnostic procedures, other methods that suffer less from the patchy distribution of eosinophils, including flow cytometric evaluation, should be incorporated in future study designs in order to accurately quantify immune cell infiltration. Finally, comparative studies should address the reproducibility of eosinophil counts across the length of the duodenum, preferably including multiple biopsies from each duodenal subsection.

Another caveat arises when comparing the low-grade immune activation in FD to the substantial inflammation found in EGID such as eosinophilic gastroenteritis. Besides a more pronounced increase in eosinophil numbers, also increased duodenal and colonic levels of IL-3, -5 and GM-CSF were found in eosinophilic gastroenteritis (Desreumaux et al., 1996), a finding not confirmed in FD. Nevertheless, certain mechanisms underlying eosinophil infiltration could be shared by EGID and DGBI, or both conditions may represent two ends of the same immune-mediated disease spectrum.

With only limited evidence supporting a Type 2 mediated infiltration of eosinophils in FD, other pathways cannot be excluded. Eosinophils were recently proposed as crucial effectors of the IL-23-GM-CSF axis in colitis with a Type 17 recruitment of eosinophils *via* GM-CSF following IL-23 release *via* antigen-presenting cells (Griseri et al., 2015). Preliminary data suggested that the Th17.1 population was increased in both the circulation and the duodenal mucosa of FD patients, indicative of auto-immune type immune reactions (Burns et al., 2021).

Non-classical eosinophil inducers include epithelial-derived cytokines thymic stromal lymphopoietin (TSLP), IL-25 and IL-33, which activate eosinophils as part of a Type 2 allergic response, often in a cooperative manner and involving ILC2 induction (Kim et al., 2013; Salimi et al., 2013; Han et al., 2017, 2018). A novel epithelial regulator of eosinophil recruitment was recently discovered in a mouse model of allergic rhinitis where protein arginine methyltransferase 1 (PRMT1) promoted the production of TSLP, IL-25 and IL-33 (Park et al., 2021). However, we found no difference in gene-expression of aforementioned cytokines in FD patients compared to healthy controls, suggesting that this pathway is not an essential contributor to eosinophilic infiltration in FD (Ceulemans et al., 2021).

## Conclusion

Research in the field of DGBI is rapidly evolving, and although the presence of intestinal immune activation in these disorders has widely been recognized, increasing evidence supports the concept of an elevated immune response as key pathophysiological event in DGBI. Increased infiltration and degranulation of both eosinophils and mast cells is frequently linked with typical GI symptoms, while treatments reducing local immune activation also yield therapeutic benefit. Immune activation should therefore be regarded as an important contributor to DGBI pathophysiology, and both direct anti-inflammatory therapies, as well as preventive or therapeutic approaches indirectly leading to reduced immune cell infiltration, should be studied further.

Furthermore, the separate events in the triad of mucosal alterations, DGBI symptomatology and psychosocial comorbidities all have been demonstrated independently, while cross-links have been made by associations between those factors. Nevertheless, describing the cause-consequence relationship between the three aforementioned manifestations continues to be one of the major challenges in DGBI research and should have the highest priority in future studies.

## Author Contributions

MC wrote the original draft. All authors contributed to the manuscript, revised and approved the final version of the review.

## Conflict of Interest

LW reports financial support for research from Danone and MyHealth; has served on the advisory board of Naturex; has served on the Speaker bureau for Falk Pharma, Takeda and MyHealth. TV reports financial support for research from Danone, MyHealth, Takeda and VectivBio; has served on the Speaker bureau for Abbott, Falk Pharma, Fresenius Kabi, Menarini, Remedus, Takeda, Truvion and VectivBio; reports consultancy fees from Baxter, Falk Pharma, Takeda, VectivBio and Zealand Pharma. The remaining authors declare that the research was conducted in the absence of any commercial or financial relationships that could be construed as a potential conflict of interest.

## Publisher’s Note

All claims expressed in this article are solely those of the authors and do not necessarily represent those of their affiliated organizations, or those of the publisher, the editors and the reviewers. Any product that may be evaluated in this article, or claim that may be made by its manufacturer, is not guaranteed or endorsed by the publisher.

## Abbreviations

BDNF: Brain-derived neurotrophic factor
CCL: CC chemokine ligand
CCR: CC chemokine receptor
CI: Confidence interval
CLE: Confocal laser endomicroscopy
CRH: Corticotropin-releasing hormone
CRHR: CRH receptor
DGBI: Disorder of gut-brain interaction
EC: Enterochromaffin cell
ECP: Eosinophil cationic protein
EDN: Eosinophil-derived neurotoxin
EGID: Eosinophil-associated GI disorder
EoE: Eosinophilic esophagitis
EPO: Eosinophil peroxidase
EPS: Epigastric pain syndrome
FD: Functional dyspepsia
GBA: Gut-brain-axis
GDNF: Glial cell line-derived neurotrophic factor
GE: Gastric emptying
GI: Gastrointestinal
GM-CSF: Granulocyte-macrophage colony stimulating factor
H&E: Hematoxylin & eosin
HC: Healthy control
HPA: Hypothalamic-pituitary-adrenal
HPF: High-power field
hs-CRP: High-sensitivity C-reactive protein
IBS: Irritable bowel syndrome
ICC: Interstitial cell of Cajal
Ig: Immunoglobulin
IL: Interleukin
ILC: Innate lymphoid cell
iNOS: Inducible nitric oxide synthase
MBP: Major basic protein
MC: Mast cell
MCP: Monocyte chemoattractant protein
NCGS: Non-celiac gluten sensitivity
NGF: Nerve growth factor
OR: Odds ratio
PBMC: Peripheral blood mononuclear cell
PDS: Postprandial distress syndrome
pi: Post-infectious
PPI: Proton pump inhibitor
SMD: Standard mean difference
STAT: Signal transducer and activator of transcription
Th: T-helper
TJ: Tight junction
TNF: Tumor necrosis factor
TRPV: Transient receptor potential vanilloid.
